# Effectiveness of second-generation antipsychotics: a naturalistic, randomized comparison of olanzapine, quetiapine, risperidone, and ziprasidone

**DOI:** 10.1186/1471-244X-10-26

**Published:** 2010-03-24

**Authors:** Erik Johnsen, Rune A Kroken, Tore Wentzel-Larsen, Hugo A Jørgensen

**Affiliations:** 1 Division of Psychiatry, H aukeland University Hospital, Sandviken, Pb 23, N-5812 Bergen, Norway; 2 Centre for Clinical Research, Haukeland University Hospital, Bergen, Norway; 3 Department of Clinical Medicine, Section of Psychiatry, University of Bergen, Bergen, Norway

## Abstract

**Background:**

No clear recommendations exist regarding which antipsychotic drug should be prescribed first for a patient suffering from psychosis. The primary aims of this naturalistic study were to assess the head-to-head effectiveness of first-line second-generation antipsychotics with regards to time until drug discontinuation, duration of index admission, time until readmission, change of psychopathology scores and tolerability outcomes.

**Methods:**

Patients ≥ 18 years of age admitted to the emergency ward for symptoms of psychosis were consecutively randomized to risperidone (n = 53), olanzapine (n = 52), quetiapine (n = 50), or ziprasidone (n = 58), and followed for up to 2 years.

**Results:**

A total of 213 patients were included, of which 68% were males. The sample represented a diverse population suffering from psychosis. At admittance the mean Positive and Negative Syndrome Scale (PANSS) total score was 74 points and 44% were antipsychotic drug naïve. The primary intention-to-treat analyses revealed no substantial differences between the drugs regarding the times until discontinuation of initial drug, until discharge from index admission, or until readmission. Quetiapine was superior to risperidone and olanzapine in reducing the PANSS total score and the positive subscore. Quetiapine was superior to the other drugs in decreasing the PANSS general psychopathology subscore; in decreasing the Clinical Global Impression - Severity of Illness scale score (CGI-S); and in increasing the Global Assessment of Functioning - Split version, Functions scale score (GAF-F). Ziprasidone was superior to risperidone in decreasing the PANSS positive symptoms subscore and the CGI-S score, and in increasing the GAF-F score. The drugs performed equally with regards to most tolerability outcomes except a higher increase of hip-circumference per day for olanzapine compared to risperidone, and more galactorrhoea for risperidone compared to the other groups.

**Conclusions:**

Quetiapine appears to be a good starting drug candidate in this sample of patients admitted to hospital for symptoms of psychosis.

**Trial Registration:**

ClinicalTrials.gov ID; URL: http://www.clinicaltrials.gov/: NCT00932529

## Background

For a patient suffering from psychosis, most second-generation antipsychotics (SGAs) have been considered first-line agents based on their more favorable tolerability profiles compared with older first-generation drugs [1-4]. This particularly applies to first episode psychosis [1,3]. Most treatment guidelines are centered on schizophrenia, and the empirical evidence is very limited for non-schizophrenic psychotic disorders [5]. Differential antipsychotic efficacy of the first-line antipsychotics remains to be convincingly demonstrated despite their differing pharmacological properties. The lack of differences regarding efficacy may be caused by limitations of the evidence base. The highly selected samples and rigid experimental designs of traditional randomized, controlled trials may restrict their ability to deliver all clinically relevant information [6]. Contradictory results in studies from different sources of pharmaceutical sponsorship may also contribute to the inconclusiveness of the evidence [7].

In recent years, several studies of the effectiveness of antipsychotics have been launched to address some of the limitations associated with traditional randomized controlled trials (RCTs) of efficacy. Effectiveness trials, also known as "naturalistic", "real-life", "pragmatic", or "practical" trials, address how a treatment works under normal clinical circumstances as distinct from the somewhat artificial settings of the efficacy trials [6]. Through pragmatic designs and more global outcome measures, these trials have been expected to supplement the base of evidence regarding effectiveness of antipsychotics. The larger studies have been financially sponsored by noncommercial sources, addressing also the problem of funding bias. In a recent systematic review of randomized head-to-head comparisons of the effectiveness of SGAs, differences among the SGAs were only consistent across trials for a limited number of outcomes [8]. In patients with chronic psychosis, olanzapine patients took a longer time to discontinuation of treatment and had better treatment adherence compared with other SGAs, but this treatment was also associated with more adverse metabolic effects. The psychopathology and most tolerability outcomes were otherwise surprisingly equal among groups. However, a significant finding in the review of effectiveness trials was a very high drug discontinuation rate in a short-term perspective for all the SGAs. About three-quarters of the patients had discontinued their allocated SGA within 18 months, with a median time until discontinuation of 5.5 months as found in the CATIE study [9]. To the authors' best knowledge, trials of the comparative effectiveness of SGAs have focused solely on the period during which the patients have used their allocated drugs. By this strategy, the results remain equivocal and do not supplement the evidence base regarding effectiveness as expected. An alternative strategy would be to assess antipsychotic effectiveness in a period extended beyond use of the first-assigned drug. Given the frequently chronic nature of schizophrenia and related disorders, and taking into account the new findings on discontinuation rates, the antipsychotic drug regimen at a given time is likely to be part of a sequence of antipsychotics. The principal question in this strategy addresses which SGA should be the starting drug in order to provide the most beneficial outcome of antipsychotic treatment.

### Aims of the study

The aim of the present study was to assess antipsychotic effectiveness in a period extending beyond the use of the first drugs.

## Methods

### Study design

The Bergen Psychosis Project (BPP) is a 24-month, prospective, rater-blind, naturalistic, randomized, head-to-head comparison of the effectiveness of olanzapine, quetiapine, risperidone, and ziprasidone. All patients were recruited from the Division of Psychiatry at Haukeland University Hospital with a catchment population of about 400000. The BPP was approved by the Regional Committee for Medical Research Ethics, and the Norwegian Social Science Data Services. Funding of the project was initiated by the Research Council of Norway, followed by Haukeland University Hospital, Division of Psychiatry. The BPP has not received any financial or other support from the pharmaceutical industry.

### Patients

The Regional Committee for Medical Research Ethics allowed eligible patients to be included before informed consent was provided, thus entailing a clinically relevant representation in the study. In medical research the provision of informed consent from the participants is fundamental. The disqualification of the most gravely ill patients from participating in trials represents an ethical dilemma; however, as these patients will most likely receive the drugs once they are approved for marketing, despite the lack of evidence from this population. Trial inclusion of patients without informed consent is justifiable on 2 conditions: That no other context exists in which the research question can be answered, and that all patients get clear clinical benefit from whatever treatment they are allocated to [10]. These criteria are fulfilled in some mental conditions from which important studies have been published [11,12]. Patients (age ≥ 18 years) were eligible for the study if they were admitted to the emergency ward for symptoms of psychosis as determined by a score of ≥ 4 on one or more of the items Delusions, Hallucinatory behavior, Grandiosity, Suspiciousness/persecution, or Unusual thought content in the Positive and Negative Syndrome Scale (PANSS) [13], and were candidates for oral antipsychotic drug therapy. Eligible patients met ICD-10 [14] diagnostic criteria for schizophrenia, schizoaffective disorder, schizophreniform disorder, brief psychotic episode, delusional disorder, drug-induced psychosis, and major depressive disorder with psychotic features. The diagnoses were determined by experienced clinicians. Patients were excluded from the study if they were unable to use oral antipsychotics, were suffering from manic psychosis, were unable to cooperate reliably during investigations, did not understand spoken Norwegian language, were candidates for electroconvulsive therapy, or were medicated with clozapine on admittance. Patients with drug-induced psychoses were included only when the condition did not resolve within a few days and when antipsychotic drug therapy was indicated.

### Treatments

The evidence thus far shows that to prospectively predict which antipsychotic might be optimal for a given patient with regards to effect and tolerability is not possible, and that antipsychotic therapy currently involves a trial and error approach [15]. A prior history of antipsychotic drug use may provide some information, though. Taking these factors into account the BPP protocol mimicked the normal clinical situation in which oral antipsychotic drug therapy is initiated, with one exception: At admission, a sealed and numbered envelope was opened by the attending psychiatrist and then the patient was offered the first drug in a random sequence of the first-line antipsychotics in Norway - olanzapine, quetiapine, risperidone, or ziprasidone. The randomization was open to the treating psychiatrist or physician and to the patient. Both the treating clinician and/or the patient could discard the SGA listed as number 1 on the list because of medical contraindications for the use of, or prior negative experiences with the drug, however, and the next on the list could be chosen. The same principle was followed if the next drug could not be used. A reason for discarding drugs was sought. In each sequence, the SGA listed as 1 defined the randomization group (RG). The actual SGA chosen, regardless of randomization group, defined the first-choice group (FCG). Further dosing, combination with other drugs, or switching to another antipsychotic drug were then left at the clinician's discretion. Apart from sporadic use, the patients in the project could use only one antipsychotic drug except during the cross-taper period associated with a change of antipsychotic drug. This is in correspondence with leading treatment guidelines which mention combinations of antipsychotics only as a last resort. In cases where concomitant use of more than one antipsychotic drug was found inevitable, the patient was excluded from the project. Any investigation that was beyond normal clinical practice was introduced only after informed consent was obtained.

### Assessments

Study visits were at baseline, at discharge or at 6 weeks from baseline at the latest, and at 3, 6, 12, and 24 months from baseline.

All assessments were performed by one trained investigator. Before inclusion, eligible patients were interviewed by the investigator, using the PANSS, the Calgary Depression Scale for Schizophrenia (CDSS) [16], and the Clinical Drug and Alcohol Use Scales (CDUS/CAUS) [17], and were rated according to the Clinical Global Impression--Severity of Illness scale (CGI-S) [18], and the Global Assessment of Functioning--Split Version, Functions scale (GAF-F) [19]. The patients received a physical examination by the admitting physician, and standard blood samples were collected according to the hospital's routine. At discharge from the hospital or at 6 weeks if not discharged, the tests and examinations were repeated by the rater who was unaware of the treatment. Patients were asked also to complete the patient-administered version of the UKU Side Effect Rating Scale (UKU-SERS Pat) [20], and serum level measurements of the antipsychotics were conducted. Thus far, all investigations and tests were part of the hospital's routine for the management of patients suffering from psychosis and became part of the patient's medical record. At this point, the patients were asked for informed consent to be contacted and included in the follow-up project.

At follow-up visits 3, 6, 12, and 24 months after baseline, measures of psychopathology, function, and tolerability, as well as clinical and laboratory assessments were repeated by the rater blind to treatment.

The global outcomes measures were: the time until discontinuation of the initial SGA for any cause, the time until discharge from index hospitalization, and the time until readmittance to the emergency ward for any reason. Symptoms were assessed by the PANSS, the CDSS, the CGI-S, and the GAF-F. Tolerability was measured by the UKU-SERS-Pat, physical examinations, and laboratory tests. The repeated physical examinations included Body Mass Index (BMI), waist and hip circumferences, and blood pressure. Laboratory tests included electrocardiogram (ECG) and blood tests on glucose, lipids, prolactin, and liver functions. The patients were fasting before the drawing of blood, as defined by no intake of food or caloric drink during the preceding 9 hours.

At each visit, all medications were recorded, and the mean antipsychotic drug doses were calculated. Antipsychotic drug doses for antipsychotics other than the SGAs were converted to chlorpromazine equivalent doses [21]. In cases were chlorpromazine equivalent doses could not be found in the literature, this was done by conversion to defined daily doses (DDDs) as developed by the World Health Organization Collaborating Centre for Drug Statistics Methodology [22]. The basic definition of the DDD unit is the assumed average maintenance dose per day for a drug used for its main indication in adults.

### Statistical procedures

The primary analyses were intention-to-treat (ITT) analyses based on the randomization groups (RGs), that is trial participants were analyzed in the group to which they were randomized regardless of which treatment they actually received or how much treatment they received [23]. Secondary analyses were based on first choice groups (FCGs). Baseline data of FCGs were analyzed using SPSS software, version 15 (SPSS, Chicago, IL), and by means of exact χ^2 ^tests for categorical data and one-way ANOVAs for continuous data. For multiple comparisons, Benjamini-Hochberg adjustments were applied. For continuous data that were not approximately normally distributed, a Kruskal-Wallis nonparametric test was used. For baseline comparisons between those lost to follow-up before retesting and those who were retested, independent samples T-tests were used for continuous data and exact χ^2 ^tests for categorical data.

Global outcomes were analyzed using SPSS, version 15, with Kaplan-Meier analyses of survival. Change of symptoms and tolerability outcomes were analyzed in R by means of linear mixed effects (LME) models [24,25]. Fixed effects, i.e. systematic differences between the drugs, were different linear slopes in the four treatment groups, technically a group by time interaction with no baseline group differences. The model calculates overall change per time unit for the variables in the follow-up period that can be visually represented by the slope of a linear curve with time on the *x *axis and the respective variable on the *y *axis. The target of the present study was to investigate the *over-all *change during the follow-up period and the LME model was considered the analysis of choice for this purpose. The model uses all available data and handles different numbers of visits by individual patients, as well as differences in times between visits. Furthermore, the mixed effects model has demonstrated superior statistical power when the missing data is non-ignorable [26]. A linear slope for the follow-up period may represent an over-simplification, however, as it does not capture slope differences at different times. Based on results from other effectiveness studies symptom changes typically follow an initial steep decline followed by a flatter curve [9,27]. LME sensitivity analyses were therefore undertaken separately for the steep and for the flat part of the symptom curves. The choice of period corresponding to the steep and flat part was derived from visual information from plots of the individual symptom curves. The draw-back of dividing the follow-up is loss of statistical power and hence risks of statistical type II errors.

Symptom ratings, laboratory tests and physical examinations were administered on all visits. The UKU-SERS-Pat was administered at visit 2 and following visits. Because differences between treatment groups on UKU-SERS-Pat measures could theoretically be present at visit 2, this was allowed for in the statistical model. For multiple comparisons, Benjamini-Hochberg adjustments were applied. The level of statistical significance was set at α = 0.05.

## Results

**Figure 1:**
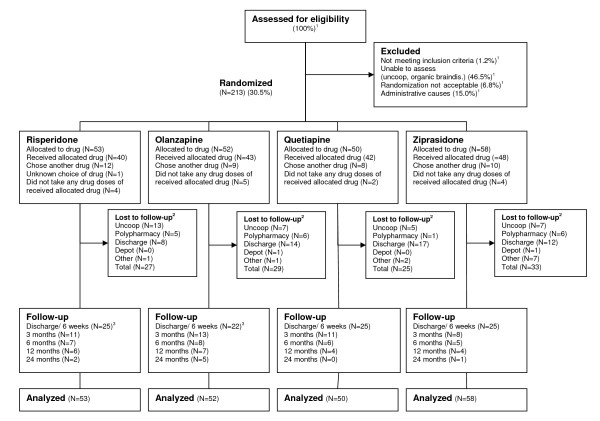
**Flow of patients through the study**. Not meeting inclusion criteria = Score below 4 on all the items Delusions, Hallucinatory behaviour, Grandiosity, Suspiciousness/persecution, or Unusual thought content in the Positive and Negative Syndrome Scale (PANSS); Uncoop. = the patient was not able or willing to cooperate with testing and assessments; Organic braindis. = Organic brain disorder, principally dementia; Randomization not acceptable = patient or treating clinician not willing to change existing antipsychotic medication; Administrative causes = principally patient discharged before assessments could be made. ^1 ^Enrolment started March 2003 until 2008, week 26. Full details on enrolment were only registered from 2006, week 31 until 2008, week 26. Consequently only percentages are displayed for patients assessed for eligibility and excluded patients. ^2 ^Before discharge/6 weeks. ^3 ^One patient in the risperidone and olanzapine groups missed the first follow-up visit, but was retested on later visits.

The patient enrolment is displayed in Figure 1. A total of 213 patients were allocated to randomized sequences of the first-line SGAs listed from 1 to 4. The SGAs listed as 1 defined the randomization groups (RGs). A total of 173 (81.2%) patients received the SGA listed as 1, whereas 39 (18.3%) chose another SGA on the list. The choice of SGA was unknown for one patient. There were no differences among RGs in the fractions of patients that did not choose the SGA listed as 1.

### Primary outcomes - ITT analyses based on RGs

Baseline demographic and clinical characteristics are presented in Additional file 1. There were no substantial differences between the randomization groups regarding proportions with life-time antipsychotic drug exposure, or proportions that had used antipsychotic drugs in the 12 months prior to admittance or in the antipsychotic agents used in that period. There were generally no substantial differences on baseline clinical or demographic characteristics between those who were lost to follow-up before retesting and those who were retested, with the exception of a slightly higher PANSS negative subscore for those lost to follow up (20.9 vs. 18.1 points (independent samples T-Test: p = 0.007; mean difference 2.75 points; 95% confidence interval (CI) 0.74-4.75)).

#### Global outcomes

**Figure 2:**
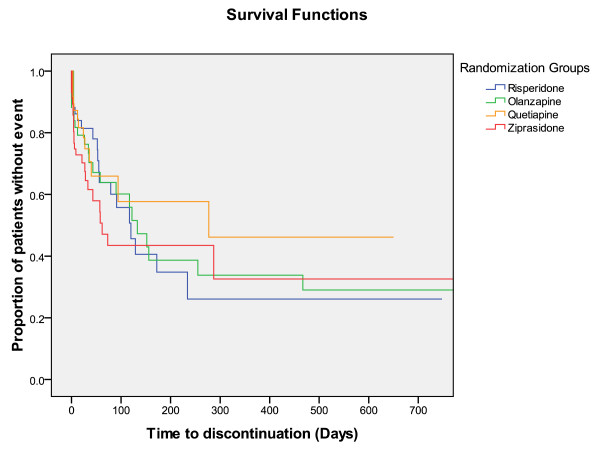
**Survival functions**. Time to discontinuation = Time (days) until discontinuation of first antipsychotic drug since index admission.

**Figure 3:**
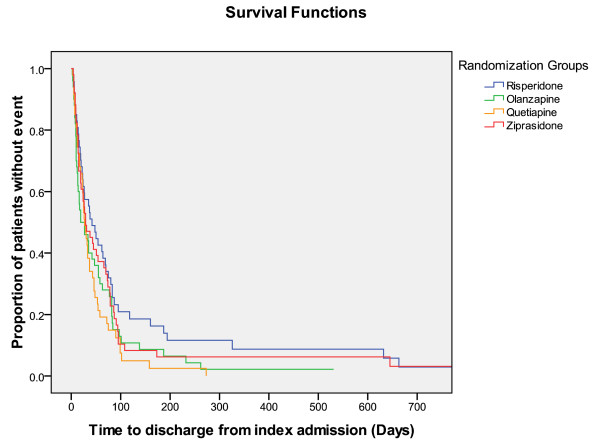
**Survival functions**. Time to discharge from index admission = Time (days) until hospital discharge after index hospital admission.

**Figure 4:**
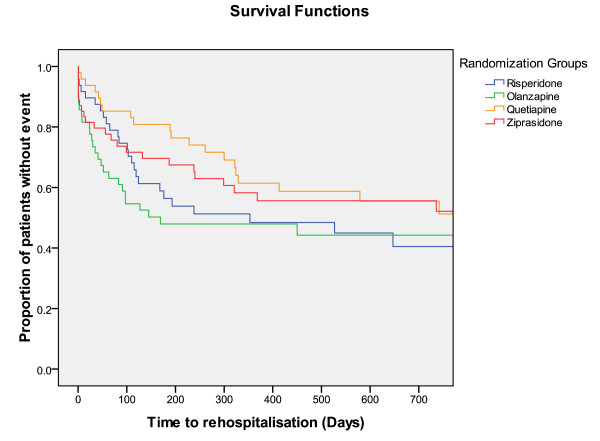
**Survival functions**. Time to rehospitalisation = Time (days) until rehospitalisation after discharge from index admission.

Times until discontinuation of the first offered antipsychotic drug, until discharge from index admission, and from discharge from index admission until readmission, were not different among RGs (Figures 2, 3, and 4).

#### Symptom outcomes

**Figure 5:**
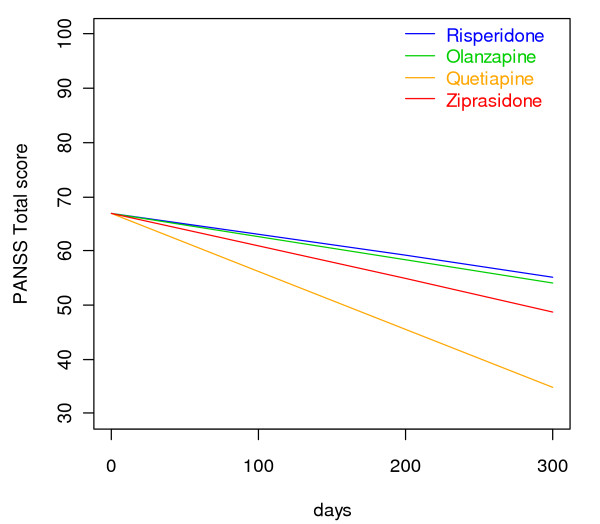
**Reduction of PANSS total score. Linear mixed effects model curves**. Linear slopes for the randomization groups generated based on linear mixed effects models PANSS total score output as displayed in Additional file 2 for risperidone, olanzapine, quetiapine, and ziprasidone, respectively. The curves are confined to the first 300 days because the major bulk of data is obtained before 300 days.

**Figure 6:**
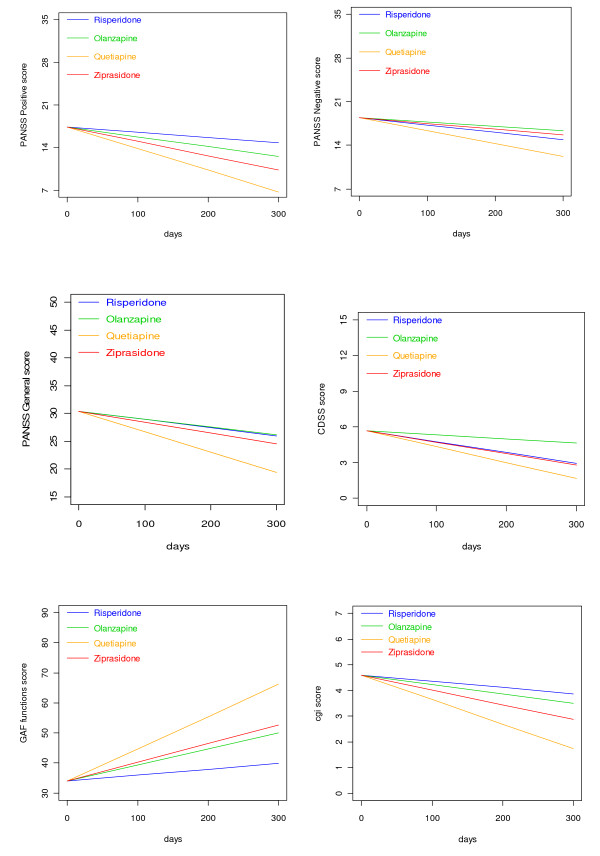
**Change of PANSS subscores, CDSS, GAF-F, and CGI-S scores**. The curves are generated based on drug-specific linear mixed effects slopes as displayed in Additional file 2 for risperidone, olanzapine, quetiapine, and ziprasidone, respectively. PANSS = the Positive and Negative Syndrome Scale; CDSS = the Calgary Depression Scale for Schizophrenia; GAF-F = the Global Assessment of Functioning scale - Split Version, Functions scale; CGI-S = the Clinical Global Impression - Severity of Illness Scale. The curves are confined to the first 300 days because the major bulk of data is obtained before 300 days.

Outcomes related to symptom reduction and increased functioning are shown in Additional file 2 and Figures 5 and 6. There were significant differences among SGAs as quetiapine was superior to risperidone and olanzapine in reducing the PANSS total score and the positive subscore. Quetiapine was superior to the other drugs in decreasing the PANSS general psychopathology subscore; in decreasing the CGI-S; and in increasing the GAF-F score. Ziprasidone was superior to risperidone in decreasing the PANSS positive symptoms subscore and the CGI-S score, and in increasing the GAF-F score. Curves for each individual regarding the PANSS total score revealed a steeper decline initially as compared to later in the follow-up period (curves not shown). Curves for each individual on the other outcomes followed the same general pattern, with the slope being steepest initially (curves not shown). The sensitivity analyses in separate follow-up periods were performed from baseline to 90 days, corresponding to the steep part of the course, and after 90 days, corresponding to the flatter part of the course. The analyses revealed trends for the RGs that were essentially similar to the findings for the whole 2-year follow-up (data not shown). Before 90 days quetiapine and ziprasidone were superior to risperidone in increasing the GAF-F score (LME: p < 0.05, unadjusted for multiple comparisons), and quetiapine was superior to risperidone in reducing the CGI-S score (LME: p < 0.05, unadjusted for multiple comparisons). The differences were no longer statistically significant after adjusting for multiple comparisons.

Sensitivity analyses that adjusted for numerically higher proportions of antipsychotic naïve patients in the quetiapine and ziprasidone RGs, revealed essentially identical results with regards to symptom reduction and increased functioning. Sensitivity analyses that excluded patients with drug-induced psychoses revealed essentially identical results with regards to symptom reduction and increased functioning.

#### Tolerability outcomes

There were differences among the drugs for only a limited number of tolerability outcomes (Additional file 3).

### Secondary outcomes - analyses based on FCGs

There were generally no substantial differences among FCGs on baseline demographic and clinical characteristics with the exception of a slightly higher PANSS positive subscore for olanzapine (21.3 points) compared with risperidone (18.5 points) (one-way ANOVA: p = 0.007; mean difference 2.8 points; 95% CI -5.0- -0.5). The mean doses in milligrams per day with standard deviations (SD) were 3.3 (1.2) for risperidone, 14.5 (5.2) for olanzapine, 357.0 (187.2) for quetiapine, and 101.3 (44.7) for ziprasidone treated groups. The mean serum levels in nanomoles per liter with SD were 82.4 (56.9) for risperidone, 102.4 (75.1) for olanzapine, 419.7 (544.9) for quetiapine, and 173.8 (81.4) for ziprasidone. The reference ranges were 30-120, 30-200, 100-800, and 30-200 for risperidone, olanzapine, quetiapine, and ziprasidone, respectively. A total of 24 (24.7%) patients changed their first-chosen SGA during follow-up. There were no differences among the FCGs in the rates of change or choice of new antipsychotic drug. One or more doses of low-potency first-generation antipsychotics were given to 15 patients (15.8%). There were no differences among the FCGs in the number of patients receiving additional antipsychotics or the mean daily additional antipsychotic dose in chlorpromazine equivalents. Seventy-one (74.7%), 23 (24.2%), and 7 (7.4%) patients received additional benzodiazepines, antidepressants, and mood stabilizers, respectively. In 30 (39.5%) of these patients 2 or more of the additional psychotropics were used in combinations. There were no differences among FCGs in the use of these additional psychotropics. Anticholinergics were prescribed for 6 (27.3%) of risperidone treated FCGs. The corresponding figures were 1 (3.8%) for olanzapine, 0 for quetiapine, and 3 (13.0%) for ziprasidone-treated FCGs (exact χ^2 ^test: p = 0.010). There were no differences among FCGs in the rates of users of antipsychotics the year prior to index hospitalization.

#### Global outcomes

The time until discontinuation of the initially chosen SGA was significantly different among FCGs (log rank test: p = 0.028). In subanalyses, patients with olanzapine showed a longer time until discontinuation compared with those treated with ziprasidone (log rank test: p = 0.007), but not compared with the quetiapine and risperidone groups. Times until discharge from index admission and until readmission were not different among FCGs.

#### Symptom outcomes

Symptom reduction outcomes were not substantially different from those of the primary analyses (Additional file 2). The exception was for the ziprasidone comparisons with risperidone not being significantly different for the change of the PANSS positive subscore, the GAF-F score, and the CGI-S score. Sensitivity analyses before 90 days in the FCGs revealed trends similar to the ones from the ITT-analyses for the PANSS total and subscores, with the quetiapine group having the steepest slope, though not statistically significant. Olanzapine and quetiapine were superior to risperidone and ziprasidone in increasing the GAF-F score before 90 days (LME: p < 0.05 adjusted for multiple comparisons). Olanzapine was superior to risperidone and ziprasidone in reducing the CGI-S score (LME: p < 0.05 adjusted for multiple comparisons). In the analyses in the period after 90 days the other groups were superior to risperidone regarding increase of the GAF-F score (LME: p < 0.05 adjusted for multiple comparisons).

#### Tolerability outcomes

Baseline registrations of laboratory measures were not different in FCGs with the exception of a higher baseline prolactin level for risperidone (Mean 746.8 IU/L) compared with quetiapine (one-way ANOVA: p = 0.001; mean diffence 401.4 IU/L; 95% CI 128.1-674.6) and ziprasidone (one-way ANOVA: p = 0.017; mean difference IU/L 293.3; 95% CI 35.9-550.6). With regards to UKU-SERS-Pat outcomes the only statistically significant difference between FCGs was less decrease of sexual desire in the ziprasidone group compared to the olanzapine group (LME: p = 0.026). Regarding physical and laboratory measures the following comparisons revealed statistically significant differences (LME: p < 0.05): The ziprasidone group had the largest increase of triglycerides per day compared to the other groups. The risperidone group had larger increase of body weight per day than the olanzapine and quetiapine groups; as well as larger increase per day of BMI compared to the olanzapine group.

## Discussion

The study represents a naturalistic approach to the issue of effectiveness among first-choice SGAs and which of these should be preferred for a patient suffering from psychosis. About two-thirds were males, fifty-three percent represented first-time admittances, and 44% were antipsychotic drug-naïve. The mean PANSS total score at baseline was 74, range 51-110. The sample thus represents a heterogeneous group of patients with psychosis. The mean daily doses of the SGAs were in the lower end of the therapeutic range with large standard deviations, probably reflecting the relatively high proportion of drug-naïve patients who in general respond to lower doses of antipsychotic drugs.

### Global outcomes

The SGAs performed equally in the ITT analyses regarding times until discontinuation of the first offered antipsychotic drug, until discharge from index admission, and until readmission. Olanzapine-treated FCGs showed a significantly longer time to discontinuation compared with the ziprasidone-treated FCGs in the secondary analyses. Superior drug survival or better adherence for patients treated with olanzapine was also found in the systematic review on head-to-head effectiveness of SGAs, but only in chronic patients [9,28-30]. In one study on chronic patients who had discontinued perphenazine, both olanzapine and quetiapine groups had significantly longer time until treatment discontinuation than risperidone [30]. In the EUFEST study comparing haloperidol with SGAs in first-episode psychosis differences in all-cause discontinuation risk were lower with amisulpride, olanzapine, quetiapine, and ziprasidone, compared with haloperidol [27]. Because our sample consisted of both first-episode and chronically ill patients it seems reasonable that our results regarding drug survival was intermediate between those from chronic phase and first-episode studies. Alternatively the limited N in our study could represent a risk of a statistical type I error because of inadequate power, and we may accordingly have missed further differences among the groups.

### Symptom reduction

The outcomes for symptom reduction were unexpected. Quetiapine was consistently superior for all outcomes except reduction of PANSS negative symptoms and depressive symptoms according to CDSS. The mean CDSS baseline score was rather low, however. The results were similar for both RGs and FCGs, and their validity is further strengthened by the inherent consistency among outcomes on different rating scales, and that similar trends were found in supplemental analyses before and after 90 days. The latter analyses only revealed a few statistically significant differences between drugs, probably because of reduced statistical power in the supplemental analyses. To the authors' best knowledge, this is the first effectiveness study to show such differences among SGAs. In the systematic review on antipsychotic effectiveness the SGAs performed equally regarding their ability to alleviate symptoms of psychosis in all the acute phase studies including studies on first-episode patients, and in all but one chronic phase study [8,28-40]. The latter study found olanzapine to be superior to quetiapine in chronic schizophrenia patients that had previously discontinued an SGA because of intolerability [29]. In one study quetiapine performed better than risperidone on depression outcomes [36]. In the EUFEST study there were no differences between the treatment groups with regards to the PANSS and CDSS scores [27]. There were significant differences for the CGI and GAF scores, and amisulpride had the most favorable and haloperidol the least favorable outcomes in this regard. In the CUtLASS study comparisons between FGAs versus SGAs revealed no differences between the groups with regards to the PANSS, GAF, and CDSS scores [41]. In our study the quetiapine and ziprasidone treated RGs had higher percentages of antipsychotic drug naïve patients, defined as having no life-time exposure to antipsychotic drugs, at baseline compared to the other groups. Hypothetically, this could influence the results as the response to antipsychotics is usually better for first episode patient compared to chronic multi-episode patients. The differences between groups regarding fractions of antipsychotic drug naïve patients were not statistically significant, however, and additional sensitivity analyses revealed essentially the same results. We have not been able to find any differences in baseline demographic or clinical characteristics that could introduce a systematic bias to the results. In the secondary analyses based on FCGs the only significant difference among the drugs was a slightly higher PANSS positive score for the olanzapine group at baseline. As the outcome measure is reduction of PANSS positive score per day, the expected bias could actually be in favor of olanzapine as a higher baseline score has a higher potential for decrease. One could argue that given the naturalistic design with assessments not restricted to the time frame of actual use of the first SGA, the outcomes may not be related to that particular SGA but to subsequent medications. We have, however, demonstrated that about three-quarters of the patients did not change their original SGA, and that there were no differences among groups in the rate of antipsychotic medication changes or the choice of a new antipsychotic agent for those who did change. Furthermore, time until discontinuation was generally the same for all SGAs with the exception of olanzapine- versus ziprasidone-treated FCGs. Nor were there any differences in prescription rates of concomitant benzodiazepines, antidepressants, additional antipsychotics, or mood stabilizers.

### Tolerability

The outcomes for tolerability were generally the same across groups. This is consistent with the findings of other effectiveness studies in which the SGAs performed equally on most tolerability outcomes [9,28-40]. The most consistent difference between the SGAs across studies in the systematic review where related to weight gain and adverse influence on cholesterol and triglyceride levels [8]. In the EUFEST study there were only differences between haloperidol, amisulpride, olanzapine, quetiapine, and ziprasidone with regards to akathisia, parkinsonism, weight gain from baseline, and hyperprolactinemia [27]. Whereas there were no significant differences between the drugs with regards to sexual dysfunction, prevalence of overweight, weight gain > 7% from baseline, proportions with hyperglycemia, hypercholesterolemia, low HDL concentration, high LDL concentration, triglyceride concentration, or change from baseline of these metabolic variables, change from baseline of fasting insulin, or proportion with QTc interval prolongation [27]. In the CutLASS study no significant differences were found between FGA and SGA groups [41]. The results may suggest that clear-cut side-effect profiles from premarketing RCTs are less distinct in a naturalistic setting where samples are more heterogeneous and concomitant medications less restricted. Our secondary outcomes on metabolic effects were somewhat surprising as the olanzapine-treated FCG was the only group that had a reduction of triglycerides, and even though patients in all FCGs gained weight and BMI, olanzapine-treated patients did so to a lesser degree than those in the other groups. One explanation may be that there is a high awareness among clinicians of olanzapine-associated metabolic adverse effects and that patients at risk of massive weight gain were identified very early and changed to another antipsychotic agent. Obviously, interactions with concomitant psychotropic drugs may also confuse the picture. The finding of equality among FCGs regarding neurological side effects must be interpreted bearing in mind that there was a significant difference among FCGs in the use of anti-cholinergic drugs, with the risperidone-treated patients having the highest rate of anti-cholinergic prescriptions. The finding of equality among FCGs regarding prolactin elevation should also be interpreted in light of the significantly higher baseline prolactin level in risperidone-treated patients compared with patients in both quetiapine-and ziprasidone-treated groups. The outcomes for autonomic side effects should be interpreted with caution because of their borderline internal reliability.

### Limitations

Some limitations to the study need to be discussed. The randomization was open to both the treating clinician and the patient. Systematic utilization differences among the SGAs before the start of the study could theoretically have introduced bias if some of the SGAs under investigation were associated with more prior experience among the RGs. The direction of such theoretical bias is hard to predict, as both negative and positive prior experiences could influence the attitude towards the SGAs under investigation. We registered prior life-time use of antipsychotic drugs as a "yes" or "no" variable. The registration of antipsychotic drug use in the 12 months prior to study inclusion was limited to whether or not antipsychotics were used, and if so, which antipsychotic drug was used. Even though more detailed information on the duration of this treatment would have added value to the paper; that was not the primary target of the study. There were, however, no substantial differences between the RGs regarding proportions with life-time exposure to antipsychotic drugs or with regards to the agents used in the 12 months prior to inclusion. Furthermore, there were no substantial differences between the randomization groups regarding the proportion who accepted the SGA listed as 1. Theoretically, the open design could also introduce bias if some of the SGAs were more popular among the clinicians or patients. However, we have no clear indications of any trends in the choices of SGAs or later changes.

Even though the exclusion criteria were limited compared with those in RCTs for efficacy, the sample represents only about 30% of those assessed for eligibility which could be a source of selection bias. Others have found the proportion of patients included in clinical trials to be in the range of 7%-14% of those initially assessed [42-44]. At least at the lower end of this range, the inference of trial results to the whole population under investigation can be questioned.

There was a high attrition rate in the study, which could be a source of bias if attrition was significantly different in the treatment groups. However, we found no significant difference in total attrition between treatment groups, and total attrition was not significantly related to baseline characteristics. Also, the primary analyses were ITT analyses based on the randomization groups.

For some of the outcome measures, the sample size may have been too small to detect actual differences among the drugs, resulting in type II errors. This may particularly be true for the survival outcomes. In addition the high rate of dropouts through follow-up led to much censoring resulting in less statistical power. The symptom outcomes and tolerability outcomes are less vulnerable because of the statistical method used.

The naturalistic design aspires to mimic clinical practice in which the antipsychotic treatment is initiated before the diagnosis for a particular patient is specified although for some of the SGAs this represents off-label use. Patients were included consecutively because of psychosis per se, and diagnostic evaluations were performed later by the treating clinicians. Accordingly, the sample is heterogeneous with respect to diagnoses and duration of the psychotic disorder among others, and there were insufficient statistical power to perform secondary analyses in subgroups which limits the inference of trial results to selected sub-populations suffering from psychosis.

Finally, the CDSS was used to assess symptoms of depression. The CDSS is primarily developed to assess depression in patients with schizophrenia, and may not be the optimal tool in assessing depressive features in other diagnostic categories.

## Conclusions

In this heterogeneous sample of patients acutely admitted to hospital for symptoms of psychosis, the quetiapine group was associated with the most beneficial outcome in terms of reduction of the PANSS total score; the PANSS positive subscale score; the PANSS general psychopathology subscale score; the CGI-S score; and in increasing the GAF-F score. There were no substantial differences among the SGAs regarding tolerability outcomes.

## Competing interests

Funding of the project was initiated by the Research Council of Norway, followed by Haukeland University Hospital, Division of Psychiatry. The supporters had no role in the design and conduct of the study; collection, management, analysis, and interpretation of the data; or preparation, review or approval of the manuscript.

EJ has received honoraria for lectures given in meetings arranged by Bristol-Myers Squibb, Eli Lilly, and AstraZeneca, and for a contribution to an information brochure by Eli Lilly.

## Authors' contributions

EJ drafted the manuscript and collected the data. RK and HAJ helped drafting the manuscript and participated in the data collection. TWL helped drafting the manuscript, provided statistical analyses and made substantial contributions to the analysis and interpretations of the data. All authors read and approved the final manuscript.

## Pre-publication history

The pre-publication history for this paper can be accessed here:

http://www.biomedcentral.com/1471-244X/10/26/prepub

## Supplementary Material

Additional file 1 **Table S1. Baseline demographic and clinical characteristics**. Baseline demographic and clinical characteristics of the Bergen Psychosis Project sample. Click here for file

Additional file 2 **Table S2. Symptoms outcomes**. Comparisons between risperidone, olanzapine, quetiapine, and ziprasidone groups with regards to change of the scores of the Positive and Negative Syndrome Scale scores; the Calgary Depression Scale for Schizophrenia; the Global Assessment of Functioning scale - Split version, Functions scale; and the Clinical Global Impression - Severity of Illness scale. Click here for file

Additional file 3 **Table S3. Tolerability outcomes**. Comparisons between risperidone, olanzapine, quetiapine, and ziprasidone groups with regards to change of side effects and tolerability outcomes. Click here for file
