# Supplementary material for: Effectiveness of second-generation antipsychotics: a naturalistic, randomized comparison of olanzapine, quetiapine, risperidone, and ziprasidone
Source: BMC Psychiatry. 2010 Mar 24;10:26. doi: 10.1186/1471-244X-10-26 (PMC2851682; doi:10.1186/1471-244X-10-26)
Supplement: Additional file 1 — Table S1. Baseline demographic and clinical characteristics. Baseline demographic and clinical characteristics of the Bergen Psychosis Project sample. [file 1471-244X-10-26-S1.DOC]

Table 1 Baseline demographic and clinical characteristics.

|  | | Randomization Groups | | | | | | | | |  | |
| --- | --- | --- | --- | --- | --- | --- | --- | --- | --- | --- | --- | --- |
| Characteristics | | Risperidone  (N=53) | | Olanzapine  (N=52) | | Quetiapine  (N=50) | | Ziprasidone  (N=58) | | All Patients  (N=213) | | |
|  | | N | % | N | % | N | % | N | % | N | | % |
| Gender | |  |  |  |  |  |  |  |  |  | |  |
|  | Male | 37 | 69.8 | 33 | 63.5 | 33 | 66.0 | 41 | 70.7 | 144 | | 67.6 |
| Ethnicity | |  |  |  |  |  |  |  |  |  | |  |
|  | White | 46 | 86.8 | 48 | 92.3 | 48 | 96.0 | 52 | 89.7 | 194 | | 91.2 |
|  | Other | 7 | 13.2 | 4 | 7.7 | 2 | 4.0 | 6 | 10.3 | 19 | | 8.8 |
| Antipsychotic naive | | 19 | 35.8 | 18 | 34.6 | 27 | 54.0 | 29 | 50.0 | 93 | | 43.7 |
| First admission | | 27 | 50.9 | 27 | 51.9 | 28 | 56.0 | 31 | 53.4 | 113 | | 53.1 |
| Alcohol last 6 mths | |  |  |  |  |  |  |  |  |  | |  |
|  | None | 15 | 28.3 | 10 | 19.2 | 8 | 16.0 | 10 | 17.5 | 43 | | 20.3 |
|  | Misuse | 2 | 3.8 | 5 | 9.6 | 10 | 20.0 | 5 | 8.6 | 22 | | 10.4 |
| Drugs last 6 mths | |  |  |  |  |  |  |  |  |  | |  |
|  | None | 32 | 61.5 | 38 | 73.1 | 36 | 72.0 | 38 | 66.7 | 144 | | 68.2 |
|  | Misuse | 11 | 21.2 | 9 | 17.3 | 7 | 14.0 | 11 | 19.3 | 38 | | 18.0 |
| Diagnosis^1^ | |  |  |  |  |  |  |  |  |  | |  |
|  | Schz and rel. | 26 | 52.0 | 20 | 40.0 | 22 | 44.0 | 22 | 40.0 | 90 | | 43.9 |
|  | Acute | 7 | 14.0 | 13 | 26.0 | 12 | 24.0 | 12 | 21.8 | 44 | | 21.5 |
|  | Drug-induced | 8 | 16.0 | 6 | 12.0 | 7 | 14.0 | 6 | 10.9 | 27 | | 13.2 |
|  | Affective | 4 | 8.0 | 6 | 12.0 | 6 | 12.0 | 6 | 10.9 | 22 | | 10.7 |
|  | Rest | 5 | 10.0 | 5 | 10.0 | 3 | 6.0 | 9 | 16.4 | 22 | | 10.7 |
|  |  | Mean/Median | SD/Range | Mean/Median | SD/Range | Mean/Median | SD/Range | Mean/Median | SD/Range | Mean/Median | | SD/Range |
| Age | | 34.4/ 30.0 | 13.3/ 18-67 | 32.2/ 27.0 | 12.2/ 18-72 | 36.9/ 36.0 | 13.9/ 18-72 | 34.3/ 31.0 | 14.8/ 17-73 | 34.4/ 31.0 | | 13.6/17-73 |
| PANSS Total | | 74.2/ 74.0 | 13.2/ 51-110 | 75.0/ 73.0 | 14.3/ 44-11 | 74.4/ 72.0 | 14.2/ 47-111 | 71.3/ 71.0 | 12.2/ 45-100 | 73.7/ 73.0 | | 13.4/ 44-111 |
| PANSS Positive | | 19.0/18.0 | 4.7/ 12-32 | 20.7/ 21.0 | 4.5/ 11-32 | 20.2/ 20.0 | 4.1/ 12-32 | 19.5/ 19.0 | 4.6/ 11-31 | 19.8/ 20.0 | | 4.5/ 11-32 |
| PANSS Negative | | 21.4/ 22.0 | 7.6/ 7-39 | 18.7/ 16.5 | 7.8/ 7-38 | 19.8/ 18.5 | 7.2/ 7-33 | 18.7/ 17.0 | 7.2/ 8-37 | 19.6/ 19.0 | | 7.5/ 7-39 |
| PANSS General | | 33.8/ 33.0 | 6.2/ 20-51 | 35.6/ 35.5 | 7.5/ 20-54 | 34.5/ 33.0 | 7.2/ 23-56 | 33.2/ 32.5 | 5.9/ 21-50 | 34.2/ 34.0 | | 6.7/ 20-56 |
| CDSS | | 6.7/ 5.0 | 5.8/ 0-19 | 6.4/ 6.0 | 4.8/ 0-19 | 6.5/ 6.0 | 4.6/ 0-19 | 6.4/ 5.0 | 5.8/ 0-23 | 6.5/ 5.5 | | 5.3/ 0-23 |
| GAF-F | | 31.4/ 31.0 | 5.0/ 18-55 | 30.7/ 31.0 | 5.4/ 10-41 | 30.4/ 30.0 | 6.7/ 15-62 | 30.8/ 31.0 | 6.8/ 2-45 | 30.8/ 31.0 | | 6.0/ 2-62 |
| CGI | | 5.2/ 5.0 | 0.6/ 4-6 | 5.2/ 5.0 | 0.7/ 4-6 | 5.2/ 5.0 | 0.6/ 4-6 | 5.0/ 5.0 | 0.6/ 4-6 | 5.2/ 5.0 | | 0.6/ 4-6 |

Notes:

N = number of patients; SD = standard deviation; Antipsychotic naïve = No life-time exposure to antipsychotic drugs before index admission; First admission = Index admission is the first admission to a mental hospital; Misuse = Misuse or Dependence according to Mueser et al.^13^ Schz and rel. = Schizophrenia and related disorders: Schizophrenia, schizo-affective disorder, delusional disorder; Acute = Acute psychosis; Affective = Affective psychosis; Rest = Miscellaneous psychotic disorders . All diagnoses are according to ICD-10; PANSS = the Positive and Negative Syndrome Scale; CDSS = the Calgary Depression Scale for Schizophrenia; GAF-F = the Global Assessment of Functioning, split version, Functions scale; CGI = the Clinical Global Impression, severity of illness scale.

^1^ Patients with missing diagnoses are not included in list.
