# Supplementary material for: Effectiveness of second-generation antipsychotics: a naturalistic, randomized comparison of olanzapine, quetiapine, risperidone, and ziprasidone
Source: BMC Psychiatry. 2010 Mar 24;10:26. doi: 10.1186/1471-244X-10-26 (PMC2851682; doi:10.1186/1471-244X-10-26)
Supplement: Additional file 2 — Table S2. Symptoms outcomes. Comparisons between risperidone, olanzapine, quetiapine, and ziprasidone groups with regards to change of the scores of the Positive and Negative Syndrome Scale scores; the Calgary Depression Scale for Schizophrenia; the Global Assessment of Functioning scale - Split version, Functions scale; and the Clinical Global Impression - Severity of Illness scale. [file 1471-244X-10-26-S2.DOC]

Table 2 Symptoms outcomes

| Outcome Measures | | | Risperidone  (N=53) | | Olanzapine  (N=52) | | Quetiapine  (N=50) | | Ziprasidone  (N=58) |
| --- | --- | --- | --- | --- | --- | --- | --- | --- | --- |
| PANSS Total score – Change/ Day | | | -0.039 | | -0.043 | | -0.107 | | -0.061 |
| - Pairwise comparisons of treatment groups | | |  | |  | |  | |  |
|  | Risperidone - Δ Change/ Day (SE) | |  | | -0.004 (0.014) [p=0.766] | | -0.068 (0.021) [p=0.004] | | -0.022 (0.018) [0.336] |
|  | Olanzapine - Δ Change/ Day (SE) | |  | |  | | -0.064 (0.020) [p=0.004] | | -0.018 (0.016) [p=0.336] |
|  | Quetiapine - Δ Change/ Day (SE) | |  | |  | |  | | -0.047 (0.023) [p=0.083] |
| PANSS Positive score – Change/ Day | | | -0.009 | | -0.016 | | -0.036 | | -0.024 |
| - Pairwise comparisons of treatment groups | | |  | |  | |  | |  |
|  | Risperidone - Δ Change/ Day (SE) | |  | | -0.008 (0.005) [p=0.149] | | -0.027 (0.007) [p=0.001] | | -0.015 (0.006) [p=0.026] |
|  | Olanzapine - Δ Change/ Day (SE) | |  | |  | | -0.019 (0.007) [p=0.012] | | -0.008 (0.006) [p=0.174] |
|  | Quetiapine - Δ Change/ Day (SE) | |  | |  | |  | | -0.012 (0.008) [p=0.149] |
| PANSS Negative score – Change/ Day | | | -0.012 | | -0.007 | | -0.021 | | -0.009 |
| - Pairwise comparisons of treatment groups | | |  | |  | |  | |  |
|  | Risperidone - Δ Change/ Day (SE) | |  | | 0.005 (0.005) [p=0.526] | | -0.009 (0.008) [p=0.526] | | 0.003 (0.007) [p=0.718] |
|  | Olanzapine - Δ Change/ Day (SE) | |  | |  | | -0.014 (0.008) [p=0.461] | | -0.002 (0.006) [p=0.718] |
|  | Quetiapine - Δ Change/ Day (SE) | |  | |  | |  | | -0.012 (0.009) [p=0.526] |
| PANSS General score – Change/ Day | | | -0.015 | | -0.014 | | -0.037 | | -0.020 |
| - Pairwise comparisons of treatment groups | | |  | |  | |  | |  |
|  | Risperidone - Δ Change/ Day (SE) | |  | | 0.001 (0.004) [p=0.878] | | -0.022 (0.006) [p=0.002] | | -0.005 (0.005) [p=0.445] |
|  | Olanzapine - Δ Change/ Day (SE) | |  | |  | | -0.023 (0.006) [p=0.0006] | | -0.006 (0.005) [p=0.337] |
|  | Quetiapine - Δ Change/ Day (SE) | |  | |  | |  | | -0.017 (0.006) [p=0.015 ] |
| CDSS score – Change/ Day | | | -0.009 | | -0.003 | | -0.013 | | -0.010 |
| - Pairwise comparisons of treatment groups | | |  | |  | |  | |  |
|  | Risperidone - Δ Change/ Day (SE) | |  | | 0.006 (0.004) [p=0.358] | | -0.004 (0.006) [p=0.655] | | -0.000 (0.005) [p=0.932] |
|  | Olanzapine - Δ Change/ Day (SE) | |  | |  | | -0.010 (0.006) [p=0.358] | | -0.006 (0.005) [p=0.358] |
|  | Quetiapine - Δ Change/ Day (SE) | |  | |  | |  | | -0.0038 (0.0064) [p=0.655] |
| GAF-F score – Change/ Day | | | 0.020 | | 0.053 | | 0.107 | | 0.062 |
| - Pairwise comparisons of treatment groups | | |  | |  | |  | |  |
|  | Risperidone - Δ Change/ Day (SE) | |  | | 0.034 (0.010) [p=0.002] | | 0.088 (0.015) [p<0.00001] | | 0.043 (0.013) [p=0.002] |
|  | Olanzapine - Δ Change/ Day (SE) | |  | |  | | 0.054 (0.014) [p=0.001] | | 0.009 (0.012) [p=0.455] |
|  | Quetiapine - Δ Change/ Day (SE) | |  | |  | |  | | 0.045 (0.017) [p=0.009] |
| CGI score – Change/ Day | | | -0.003 | | -0.004 | | -0.010 | | -0.006 |
| - Pairwise comparisons of treatment groups | | |  | |  | |  | |  |
|  | Risperidone - Δ Change/ Day (SE) | |  | | -0.001 (0.001) [p=0.221] | | -0.007 (0.002) [p<0.00001] | | -0.003 (0.001) [p=0.021] |
|  | Olanzapine - Δ Change/ Day (SE) | |  | |  | | -0.006 (0.001) [p=0.0003] | | -0.002 (0.001) [p=0.090] |
|  | Quetiapine - Δ Change/ Day (SE) | |  | |  | |  | | -0.004 (0.002) [p=0.036] |
|  | |  | |  | |  | |  | |

Notes:

Differences with a p-value (p) < 0.05 are in bold. N = Number of Patients; Change/ Day = Mean Change of Outcome Measure per Day; Δ Change/ Day = Difference in Mean Change of Outcome Measure per Day between groups; SE = Standard Error; PANSS = the Positive and Negative Syndrome Scale; CDSS = the Calgary Depression Scale for Schizophrenia; GAF-F = the Global Assessment of Functioning, split version, Functions scale; CGI = the Clinical Global Impression, Severity of Illness scale.
