# Supplementary material for: Effectiveness of second-generation antipsychotics: a naturalistic, randomized comparison of olanzapine, quetiapine, risperidone, and ziprasidone
Source: BMC Psychiatry. 2010 Mar 24;10:26. doi: 10.1186/1471-244X-10-26 (PMC2851682; doi:10.1186/1471-244X-10-26)
Supplement: Additional file 3 — Table S3. Tolerability outcomes. Comparisons between risperidone, olanzapine, quetiapine, and ziprasidone groups with regards to change of side effects and tolerability outcomes. [file 1471-244X-10-26-S3.DOC]

**Table 3: Tolerability outcomes.**

| **Outcome Measures– Change/ Day** | | **Risperidone**  **(N=53)** | **Olanzapine**  **(N=52)** | **Quetiapine**  **(N=50)** | **Ziprasidone**  **(N=58)** |
| --- | --- | --- | --- | --- | --- |
| **Fasting Glucose (mM L)** | | -0.0001 | 0.0001 | 0.0007 | 0.0007 |
| **C-Peptide (mM/ L)** | | -0.0002 | -0.0003 | -0.0007 | -0.0006 |
| **Prolactin (IU/L)** | | -0.4181 | -0.3647 | -0.4160 | -0.5377 |
| **ALAT (U/L)** | | -0.0171 | -0.0190 | -0.0266 | -0.0056 |
| **Cholesterol – Total (mM/ L)** | | 0.0009 | 0.0007 | 0.0016 | 0.0010 |
| **Cholesterol – HDL (mM/ L)** | | 0.0001 | -0.0001 | -0.0000 | -0.0003 |
| **Cholesterol – LDL (mM/ L)** | | 0.0005 | 0.0007 | 0.0013 | 0.0004 |
| **Triglycerides (mM/ L)** | | 0.0006 | 0.0002 | 0.0018 | 0.0016 |
| **Body Weight (kg)** | | 0.0140 | 0.0170 | 0.0162 | 0.0162 |
| **Body Mass Index** | | 0.0042 | 0.0052 | 0.0058 | 0.0049 |
| **Waist Circumference (cm)** | | -0.0124 | 0.0160 | 0.0121 | 0.0161 |
| **Hip Circumference (cm)** | | -0.0102 | 0.0252 | 0.0070 | -0.0077 |
| **- Pairwise comparisons between groups** | |  |  |  |  |
|  | Risperidone - Δ Change/ Day (SE) |  | **0.0355 (0.0116) [p=0.028]** | 0.0172 (0.0085) [p=0.141] | 0.0026 (0.0193) [p=0.896] |
|  | Olanzapine - Δ Change/ Day (SE) |  |  | -0.0183 (0.0097) [p=0.141] | -0.03290 (0.0200) [p=0.164] |
|  | Quetiapine - Δ Change/ Day (SE) |  |  |  | 0.0147 (0.0183) [p=0.516 ] |
| **Rate Corrected QT interval (ms)** | | 0.0047 | -0.0074 | -0.0144 | -0.0107 |
| **Systolic Blood Pressure (mmHg)** | | -0.0033 | 0.0074 | 0.0037 | 0.0103 |
| **Diastolic Blood Pressure (mmHg)** | | -0.0074 | -0.0059 | -0.0027 | -0.0246 |
| **Psychic Side Effects** | | -0.0001 | -0.0002 | -0.0010 | -0.0005 |
| **Neurological Side Effects** | | 0.0000 | -0.0001 | -0.0009 | -0.0007 |
| **Autonomic Side Effects** | | -0.0001 | 0.0000 | -0.0007 | -0.0003 |
| **Skin Rash** | | -0.0003 | 0.0002 | 0.0004 | -0.0006 |
| **Itch** | | 0.0005 | -0.0003 | 0.0004 | -0.0005 |
| **Weight Gain** | | 0.0020 | -0.0010 | 0.0012 | 0.0026 |
| **Weight Loss** | | -0.0010 | 0.0003 | -0.0006 | -0.0003 |
| **Galactorrhoea** | | 0.0007 | -0.0001 | 0.0000 | 0.0000 |
| **- Pairwise comparisons between groups** | |  |  |  |  |
|  | Risperidone - Δ Change/ Day (SE) |  | **-0.0007 (0.0001) [p<0.001]** | **-0.0007 (0.0002) [p=0.002]** | **-0.0007 (0.0002) [p<0.001]** |
|  | Olanzapine - Δ Change/ Day (SE) |  |  | 0.0001 (0.0002) [p=0.960] | 0.0001 (0.0002) [p=0.960] |
|  | Quetiapine - Δ Change/ Day (SE) |  |  |  | 0.00000 (0.00022) [p=1.000] |
| **Gynaecomastia** | | 0.0001 | 0.0000 | 0.0001 | 0.0001 |
| **Increased Sexual Desire** | | 0.0006 | -0.0001 | -0.0001 | 0.0001 |
| **Decreased Sexual Desire** | | -0.0009 | 0.0012 | -0.0016 | -0.0016 |
| **Female Reproduction Related Side Effects** | | 0.0002 | -0.0001 | 0.0002 | 0.0089 |
| **Erectile Dysfunction** | | -0.0005 | 0.0005 | -0.0027 | -0.0004 |
| **Ejaculatory Dysfunction** | | -0.0006 | 0.0005 | -0.0010 | -0.0000 |
| **Premature Ejaculation** | | 0.0001 | -0.0001 | -0.0018 | -0.0001 |
|  | |  |  |  |  |

**Notes:**

Between-group comparisons are only displayed for statistically significant differences. Differences with a p-value (p) < 0.05 are in bold. UKU SERS-Pat = The UKU Side Effects Rating Scale, Patient Administered Version; N = Number of Patients; Change/ Day = Mean Change of Outcome Measure per Day; Δ Change/ Day = Difference in Mean Change of Outcome Measure per Day between groups; SE = Standard Error.

Psychic Side Effects = Mean sum score of the items 1.1 Concentration difficulties, 1.2 Astenia/ Lassitude/ Increased Fatigability, 1.3 Sleepiness/ Sedation, 1.4 Failing memory, 1.5 Depression, 1.6 Tension/ Inner unrest, 1.7 Increased Duration of Sleep, 1.8 Decreased Duration of Sleep, 1.9 Increased Dream Activity, 1.10 Emotional Indifference: Chronbach’s alfa 0.763;

Neurologic Side Effects = Mean sum score of the items 2.1 Dystonia, 2.2 Rigidity, 2.3 Hypokinesia/ Akinesia, 2.4 Hyperkinesia, 2.5 Tremor, 2.6 Akathisia, 2.7 Epileptic Seizures, 2.8 Paraesthesias: Chronbach’s alfa 0.810.

Autonomic Side Effects = Mean sum score of the items 3.1 Accomodation Disturbances, 3.2 Increased Salivation, 3.3 Reduced Salivation, 3.4 Nausea/ Vomiting, 3.5 Diarrhoea, 3.6 Constipation, 3.7 Micturition Disturbances, 3.8 Polyuria/ Polydipsia, 3.9 Orthostatic Dizziness, 3.10 Palpitations/ Tachycardia, 3.11 Increased Tendency to Sweating: Chronbachs alfa 0.630;

Female Reproduction Related Side Effects = Mean Sum Scores of the items 4.7a Menorrhagia, 4.7b Metrorrhagia, 4.8 Amenorrhoea, 4.15 Orgastic Dysfunction, 4.16 Dry Vagina: Chronbach’s alfa 0.708; mM = millimoles; L = Litre; IU = International Units; kg = kilo grams; cm = centimetres; U = Units; ms = milliseconds.
